# Development and validation of the Trauma-Related Cognitions Scale

**DOI:** 10.1371/journal.pone.0250221

**Published:** 2021-04-15

**Authors:** Christine E. Valdez, Melissa J. London, Steven E. Gregorich, Michelle M. Lilly

**Affiliations:** 1 Department of Psychology, California State University, Monterey Bay, Seaside, California, United States of America; 2 San Francisco VA Medical Center, San Francisco, California, United States of America; 3 School of Medicine, University of California, San Francisco, California, United States of America; 4 Department of Psychology, Northern Illinois University, DeKalb, Illinois, United States of America; Koc University School of Medicine, TURKEY

## Abstract

Cognitive theories suggest the manner in which individuals process trauma-related information influences posttraumatic sequelae. Interpretations about trauma can be maladaptive and lead to cognitive distortions implicated in the development of posttraumatic stress disorder (PTSD) through the processes of *overaccommodation* and *assimilation*. Alternatively, adaptive interpretations about trauma through the process of *accommodation* can lead to post-trauma resilience and recovery. The Trauma-Related Cognitions Scale (TRCS) provides a measure of beliefs associated with these cognitive processes. The TRCS was developed over the course of four phases. During Phase 1, 94 items derived from previously validated trauma cognition/beliefs measures were aggregated with 40 items developed by the authors. Phase 2 investigated the TRCS factor structure by fitting exploratory factor analysis (EFA) models to data from a non-clinical sample, resulting in a reduced 69-item TRCS representing four factors: the three theoretical cognitive processes of overaccommodation, assimilation, and accommodation, and an additional *optimism* factor. Phases 3 and 4 fit confirmatory factor analysis (CFA) models of the 69-item TRCS in a new non-clinical and a clinical sample, respectively, and further validation analyses were conducted. Initial evidence suggests the TRCS is a valid and reliable measure of trauma beliefs. Continued validation can determine its utility in both research and clinical contexts.

## Introduction

Most individuals experience at least one traumatic event in their lifetime [1]. Although posttraumatic stress symptoms are common reactions to traumatic events, the typical outcomes following these events are recovery or resilience [2,3]. In fact, a large study with adults in the U.S. (*N* = 2,953) estimated that posttraumatic stress disorder (PTSD) affects less than 10% of adults exposed to traumatic events [1]. Given that only a minority of trauma-exposed individuals go on to develop PTSD, continued empirical identification of factors associated with the development and/or maintenance of PTSD symptoms is warranted.

Prominent social cognitive and information-processing theories have historically recognized that the manner in which individuals process trauma-related information influences trauma-related sequelae [4–7]. Cognitive processes that have been implicated in the development of posttraumatic stress symptoms include over-accommodation and assimilation [7,8], while accommodation has been associated with recovery [9]. Accommodation involves the alteration of cognitions to incorporate new post-trauma information, and is typically associated with balanced cognitions about the traumatic event, the self, and the world (e.g., “I was hurt by another person, but that does not mean everyone is bad.”). By contrast, over-accommodation processes result in an alteration of post-trauma cognitions that are more extreme and broader in nature, such as overgeneralizing (e.g., “People are inherently evil”) or catastrophizing (e.g. “My life will never be the same”). Assimilation involves altering new information to maintain and reinforce pre-existing cognitions (e.g., “It is my fault this happened.”). These theories suggest that erroneous or extreme interpretations of the causes and/or consequences of the traumatic event interfere with recovery. Moreover, individuals may develop problematic patterns of interpreting new information based on cognitive distortions developed post-trauma. Distorted thinking may lead to avoidant behavioral and cognitive coping responses intended to reduce the sense of threat (e.g., avoiding crowds due to fear of unknown danger, constantly scanning one’s environment for threats even in relatively safe locations). However, these strategies prevent change in event-related interpretations and instead maintain posttraumatic stress symptoms such as avoidance, irritability from physiological arousal, and distress caused by intrusive thoughts and memories [10,11].

Trauma-related changes in cognitions have become explicitly recognized as diagnostic criteria important to the development and trajectory of PTSD within the most recent edition of the *Diagnostic and Statistical Manual of Mental Disorders*, *Fifth Edition* (*DSM-5*) [12]. As part of the revised diagnostic criteria of PTSD, a newly formulated symptom cluster, entitled negative alterations in cognitions and mood (NACM), was included to reflect and highlight post-trauma changes in cognitions. In particular, NACM symptoms include changes in cognitions about the self (e.g. “I am inadequate.”), others (e.g. “Everyone is malevolent.”), and the world (e.g. “The world is a dangerous place.”), as well as cognitions regarding the meaning of post-trauma symptoms (e.g. “I’m going crazy.”). Research has provided strong support for the addition of cognitive symptoms to the diagnostic criteria for PTSD [13]. Confirmatory factor analyses conducted with two large surveys [1,14] indicated that the new four cluster model of PTSD (re-experiencing; avoidance; hyperarousal and reactivity; negative alterations in cognitions and mood) provided a better fit to the data than the previous three cluster model of PTSD that excluded NACM [14]. Moreover, cognitive distortions captured within the NACM cluster have been shown to predict the development, chronicity, and severity of PTSD, as well as functional impairment associated with PTSD symptoms [10,11,15,16].

Accordingly, a central component in many empirically supported treatments for PTSD is the targeting of trauma-related cognitions. In particular, evidence has shown belief change to be one mechanism by which cognitive-behavioral treatments for PTSD have led to reductions in symptom severity [17–20]. In one of the first outcome studies examining the efficacy of Cognitive Therapy for PTSD (CT-PTSD) it was observed that reduction in negative trauma-related cognitions resulted in a reduction in PTSD symptoms over the course of CT-PTSD in a treatment-seeking community sample, though it should be noted that the cell sizes for both the intervention and waitlist conditions were quite small (*n* = 14) [21]. While trauma-related cognitions are not specifically targeted for change in Prolonged Exposure (PE) [22], a first line treatment for PTSD that indirectly challenges trauma-related cognitions, a recent study with female survivors of assault enrolled in PE showed that changes in PTSD-related cognitions predicted subsequent changes in PTSD symptoms overall; PTSD symptom reduction did not predict change in cognitions [20]. Further, a recent longitudinal study found reductions in trauma-related cognitions were related to reductions in posttraumatic stress symptoms up to 10 years after treatment in a sample of 171 women randomized to either Prolonged Exposure or Cognitive Processing Therapy treatments for PTSD [23].

Currently, measures that tap into trauma-related cognitions include the Posttraumatic Cognitions Inventory (PTCI) [24]; World Assumptions Scales (WAS) [25], Trauma and Attachment Belief Scale (TABS) [26], Trauma-Related Guilt Inventory (TRGI) [27], Trauma Appraisal Questionnaire (TAQ) [28], and Posttraumatic Maladaptive Beliefs Scale (PMBS) [29]. These measures have contributed significantly to an understanding of trauma-related cognitions involved in the trajectory of, and recovery from, PTSD symptoms. Yet, the existing measures do not assess the full range of trauma cognitions associated with all three cognitive processes discussed. That is, existing measures generally provide either information on assimilated cognitions or overaccommodated cognitions. These tools are useful in identifying erroneous and extreme cognitions, yet they fail to assess accommodated cognitions. Accommodated cognitions reflect balanced thinking about the causes and consequences of the traumatic event and may be important in assessing changes associated with recovery in trauma-focused therapy.

The primary aim of the current study was to develop a measure of cognitions consistent with the cognitive processes involved in the development and maintenance of PTSD, and one that is theory-driven and aligned with evidence-based cognitive-behavioral trauma-focused therapies. Moreover, our goal was to take a first step in developing an assessment tool that would be useful in both research and clinical contexts. A comprehensive trauma cognitions measure that taps into all three cognitive processes would be beneficial to inform treatment planning and evaluate treatment effects over time. Because research has shown that exposure to interpersonal trauma (i.e., trauma committed at the hands of another human) is the strongest correlate of cognitive symptoms of PTSD [30], the present study used samples of interpersonal trauma survivors to develop the trauma cognitions measure across the final phases of the study.

## Method

Scale construction was carried out across four phases. In the Item Generation Phase (Phase 1), the initial version of the Trauma-Related Cognitions Scale (TRCS) was created with items gathered from several validated and reliable trauma cognitions/beliefs measures in open access, as well as items generated by the first, second, and fourth author, who are all clinical research experts in trauma psychology and licensed clinical psychologists trained in trauma-focused therapy. Permission to use items from pre-existing scales was secured by the original authors of those scales. In the Exploratory Factor Analysis Phase (Phase 2), the initial version of the TRCS was administered to a non-clinical sample and an exploratory factor analysis was fit to the data to explore the factor structure and reduce TRCS items. This study was approved by Northern Illinois University’s Institutional Review Board and no adverse events were reported. In the Confirmatory Factor Analysis and Validity Assessment Phase (Phase 3), a confirmatory factor analysis (CFA) model was fit to the data from a second non-clinical trauma sample to confirm the empirical measurement model identified in Phase 2; criterion-related, convergent and discriminant, concurrent, and incremental validity were also examined. This study was approved by Northern Illinois University’s Institutional Review Board and no adverse events were reported. In Phase 4, a CFA model was fit to data from a clinical sample presenting for trauma-focused therapy at an outpatient community mental health clinic, and validity was further assessed in this sample. This study was approved by the University of California, San Francisco medical center’s Institutional Review Board and no adverse events were reported.

## Phase 1: Item generation

### Phase 1 method

#### Phase 1 measures

Items for the TRCS were chosen from trauma cognitions measures with permission from original authors granted to the research team, including the PTCI [24], WAS [25], TRGI [27], TAQ [28], and PMBS [29]. The five instruments included a total of 169 items.

The PTCI is a 36-item self-report measure of trauma-related cognitions. It contains three subscales: (1) Negative Cognitions about Self (“I am a weak person”), (2) Negative Cognitions about the World (“The world is a dangerous place”), and (3) Self Blame (“The event happened to me because of the sort of person I am”). Internal consistency coefficients in the development study ranged from *α* = .86 to *α* = .97 [24].

The WAS is a 32-item scale that measures participants’ assumptions on eight subscales, including benevolence of the world, benevolence of people, justice, controllability, randomness, self-worth, self-controllability and luck. Sample items include the following: “There is more good than evil in the world” (benevolence of the world), “People are naturally unfriendly and unkind” (benevolence of people, reverse scored) and “I have reason to be ashamed of my personal character” (self-worth). Internal consistency for the WAS in the development sample ranged from *α* = .68 to *α* = .86 [25].

The TRGI is a 32-item measure that assesses cognitive and emotional aspects of guilt associated with traumatic events. The TRGI includes a Global Guilt Scale, a Distress Scale, and a Guilt Cognitions Scale composed of three subscales: Hindsight-Bias/Responsibility (“I could have prevented what happened to me”), Wrongdoing (“I did something that went against my values”), and Lack of Justification (“What I did made sense” reverse scored). For the purposes of this study, only the 16 items from the subscales of Hindsight-Bias/Responsibility, Wrongdoing, and Lack of Justification were used. The TRGI has adequate to excellent internal consistency (α = .60 to .91), adequate temporal stability (.73 to .86), and concurrent validity [27].

The TAQ is a 54-item measure of posttraumatic appraisals that taps cognitions, emotions, and behaviors of six categories: Fear (“Danger was always present”), Betrayal (“Important people such as a parent, lover, friend let this happen to me”), Self-blame (“I must have done something really awful to make this happen”), Shame (“No shower can wash away how dirty I felt”), Alienation (“I felt lonely”), and Anger (“I felt rage”). Internal consistency ranged from .83 to .90 across three samples in the development of the TAQ, and test-retest reliability ranged from .73 to .88 across three to eight weeks [28].

The PMBS is a 15-item measure of maladaptive beliefs about current life circumstances that may occur following trauma exposure. It assesses maladaptive cognitions within three domains: Threat of Harm (“I don’t feel safe anywhere anymore”), Self-Worth and Judgment (“I am a good person”), and Reliability and Trustworthiness of Others (“Some people can be trusted”). Internal consistency for the subscales is adequate (ranging from *α* = .71 to .76) [29].

#### Phase 1 procedure

Items were selected from the five validated and reliable trauma cognitions measures if they referred to a cognition. Several items that were not selected referred to a specific feeling (e.g., PMBS: *I feel dead inside*), the cognition of the trauma at the time of occurrence (e.g., TAQ: *I didn’t know whether I would live or die*), a general opinion (e.g., WAS; *I am luckier than most people*), a behavioral fact (e.g., WAS; *I take the actions necessary to protect myself against misfortune*), or were redundant with other items already selected. In total, 59 items were not retained: 38 were dropped because of content (4 items from the PTCI, 38 items from the TAQ, 8 items from the WAS, and 2 items from the PMBS) and an additional 7 items were redundant (1 item from the TRGI, 2 items from the WAS, and 4 items from the PMBS). This procedure resulted in retention of 94 items, 11 of which were re-worded to refer to a present cognition (e.g., TAQ: *I was a bad person* was re-worded to *I am a bad person)* and to make items more concise.

From the pool of 94 items that were retained, the first, second, and fourth authors independently coded all items into one of three categories (1) overaccommodation, (2) assimilation, or (3) accommodation. The three authors then met to compare their independent codes and discuss any discrepancies until consensus was reached for which scale each item should be placed. Overaccommodation was represented by 46 items (27 items from the PTCI, 7 items from the TAQ, 12 items from the PMBS). Assimilation was represented by 38 items (15 items from the TRGI, 5 items from the PTCI, 9 items from the TAQ, 9 items from the WAS). Accommodation was represented by 10 items (7 items from the WAS, 3 items from the PMBS). After reading through item content for each subscale, it was agreed that additional items would be created to increase content validity. The first, second, and fourth authors independently created 8 provisional assimilation items and 87 provisional accommodation items. The higher number of accommodation items was created because few items from the existing scales were coded as representative of accommodative processes by the research team. After the creation of these items, the first, second, and fourth authors compared the independently created items to those from the 5 published instruments. Redundant items were discussed, and the authors came to consensus with the best wording for redundant and nonredundant items. Five items were added to the assimilation subscale and 35 items were added to the accommodation subscale. In total, the preliminary TRCS was represented by 134 items among the three subscales of overaccommodation (46 items), assimilation (43 items), and accommodation (45 items).

Finally, given the original five trauma cognitions measures had different response options, we standardized the TRCS anchors and chose six response options. Directions to complete the TRCS were modeled after the PTCI [24], and were as follows:

*We are interested in the kind of thoughts which you may have had after a traumatic experience. Below are a number of statements that may or may not be representative of your thinking*.

*Please read each statement carefully and tell us how much you AGREE or DISAGREE with each statement using the following rating scale*: 1 (*strongly disagree*), 2 (*disagree*), 3 (*somewhat disagree*), 4 (*somewhat agree*), 5 (*agree*), 6 (*strongly agree*). *People react to traumatic events in many different ways*. *There are no right or wrong answers to these statements*. Instructions were kept consistent throughout all study phases.

## Phase 2: Non-clinical sample #1 exploratory factor analysis

### Phase 2 method

#### Phase 2 participants and recruitment procedure

The Exploratory Factor Analysis Phase collected and analyzed TRCS data from a non-clinical sample. The sample included 815 participants: 509 (62.5%) community individuals recruited in 2013 through Amazon’s Mechanical Turk (MTurk) who completed the study for small payments as well as 306 (37.5%) undergraduate students from a large Midwestern university who completed the study for course credit. Studies have found MTurk samples to be more socio-economically and ethnically diverse than participants recruited via social media postings and university settings, with indistinguishable test responses across samples that complete testing online and in-person. Thus, researchers have suggested that MTurk data is as reliable as those obtained via traditional methods [31,32].

Participants age 18 and above provided electronic consent to complete the TRCS, the Traumatic Life Events Questionnaire, and other measures described below online as part of a larger study. Following the completion of the study, participants were thanked for their participation and provided a debriefing form.

The average age of participants was 27.38 (*SD* = 10.30), ranging from 18 to 67. The majority of participants identified as male (54.3%) and identified their race as Caucasian/White (59.9%); 10.3% identified as African American, 21.7% identified as Asian, 1.1% identified as American Indian or Alaskan Native, 0.8% identified as Native Hawaiian/another Pacific Islander, 2.5% identified as Biracial, 0.8% reported Unknown, and 2.8% preferred not to answer; 10.4% identified their ethnicity as Hispanic or Latino. About a quarter of participants had a college degree (24.5%), 21% reported having some high school, 16.4% reported having a high school degree/GED, 17.5% reported having some college/vocational training, 2.0% reported having some graduate training, and 14.2% reported having a graduate degree. The majority were employed full-time (35.1%) or part-time (27.1%), and were single (45.8%) or dating (19.4%). The majority of participants had experienced a potentially traumatic event (see Table 1).

**Table 1:** Rates of exposure to lifetime potential trauma in Phases 2 and 3 non-clinical samples.

Trauma Type	Phase 2 sample (*N* = 815)	Phase 3 sample (*N* = 998)
	%	%
Natural disaster	46.8	46.9
Motor vehicle accident	19.5	17.5
Other accident that caused injury	14.8	17.2
Combat experience	4.5	4.2
Unexpected death of a loved one	62.4	68.4
Loved one survived trauma	38.7	45.8
Experienced life-threatening illness	15.7	15.3
Robbed with a weapon*	11.5	13.4
Physically assaulted*	11.2	11.6
Witnessed community violence*	16.4	19.8
Threatened with injury*	34.3	24.1
Child physical abuse*	17.0	17.8
Witnessed family violence*	27.7	27.9
Intimate partner violence*	20.7	20.1
Child sexual assault by elder*	13.3	12.2
Child sexual assault by peer *	12.0	11.4
Adolescent sexual assault*	11.4	12.1
Adult sexual assault*	11.8	11.3
Unwanted sexual attention*	16.3	16.7
Stalked*	16.3	18.3
Miscarriage	12.1	11.4
Abortion	10.8	10.5
Other traumatic experience	17.3	18.0
One or more traumatic events	91.8	94.2
One or more interpersonal traumatic events	65.1	65.1

*Defined as interpersonal trauma.

#### Phase 2 measures

*Trauma cognitions*. The original 134-item TRCS was used to assess trauma cognitions.

*Trauma history*. Potential traumatic experiences were identified using the Traumatic Life Events Questionnaire (TLEQ) [33], a 23-item broad-spectrum measure of trauma exposure. Items ask respondents to identify how many times they have experienced a particular event using the following rating scale: *never* (0), *once* (1), *twice* (2), *3 times* (3), *4* times (4), *5 times* (5), and *more than 5 times* (6). In one study utilizing a clinical sample, the TLEQ demonstrated a higher rate of identification of traumatic events compared to the Structured Clinical Interview for *DSM-IV* (SCID) [34].

#### Phase 2 data analysis plan

Exploratory factor analysis models with direct oblimin (oblique) rotation were fit to the respondent data on the 134-item initial TRCS. Data were assumed missing at random and missing data were deleted listwise. Factor solutions with varying numbers of factors (i.e., 2, 3, 4, and 5) were generated and the 4-factor solution was chosen as it offered a combination of theoretical appeal and parsimony. Items with maximum absolute factor pattern loadings below .30 and items that cross-loaded (loaded on two or more factors) were dropped if the larger factor loading was less than twice as large as the lesser loading [35]. Following EFA modeling, scale scores were calculated for each factor by averaging each respondent’s corresponding item scores, such that scale scores had a possible range from 1–6. Inter-scale correlations and Cronbach alphas were estimated.

### Phase 2 results

#### Phase 2 exploratory factor analysis

The chosen exploratory factor analysis model retained 69 items representing the three hypothesized factors of Overaccommodation (e.g., "I can’t deal with even the slightest upset"; "My life has been destroyed by the trauma"; 25 items; *α* = .97), Assimilation (e.g., "I blame myself for what happened"; "What I did was inconsistent with my beliefs"; 13 items; *α* = .94), and Accommodation (e.g., "Life is sometimes a gamble"; "Life is about surviving challenging events"; 15 items, *α* = .89). In addition, an Optimism factor emerged that was not hypothesized, but made substantive sense (e.g., "The world is a good place"; "Most people are basically caring"; 16 items; *α* = .93; Table 2).

**Table 2:** Standardized factor loadings from EFA and CFA models fit to non-clinical and clinical samples.

	Non-clinical samples		Clinical sample
	Phase 2 69-item FA	Phase 3 69-item CFA	Phase 4 69-item CFA
**Over-Accommodation**			
3. I have no future. (PTCI)	.76	.67	.66
6. My life has been destroyed by the trauma. (PTCI)	.66	.71	.71
21. I am a weak person. (PTCI)	.73	.64	.55
27. I am inadequate. (PTCI)	.68	.69	.63
39. My reactions since the event mean that I am going crazy. (PTCI)	.58	.81	.66
42. I have lost my sense of freedom. (PMBS)	.60	.77	.47
45. I am a bad person. (TAQ)	.74	.74	.71
48. I will not be able to control my emotions, and something terrible will happen. (PTCI experimental item)	.64	.78	.59
49. Important people (such as parents, partner, friend) let this happen to me. (TAQ)	.41	.66	.40
51. It’s as if my insides are dirty. (TAQ)	.50	.77	.60
54. I can’t deal with even the slightest upset. (PTCI)	.67	.75	.71
63. Nothing good can happen to me anymore. (PTCI)	.77	.82	.57
66. If I think about the event, I will not be able to handle it. (PTCI)	.44	.77	.50
69. I can’t trust that I will do the right thing. (PTCI)	.58	.64	.53
75. I used to be a happy person, but now I am always miserable. (PTCI)	.67	.78	.71
78. I have permanently changed for the worse. (PTCI)	.64	.82	.77
87. There is something wrong with me as a person. (PTCI)	.80	.81	.66
90. I am not safe. (TAQ)	.79	.74	.13
93. I will never be able to feel normal emotions again. (PTCI)	.71	.81	.70
99. My reactions since the trauma show that I am a lousy coper. (PTCI)	.48	.84	.60
102. I lost my sense of manhood or womanhood. (TAQ)	.53	.81	.41
108. No shower can wash away how dirty I feel. (TAQ)	.42	.75	.64
111. I will not be able to control my anger and will do something terrible. (PTCI)	.63	.77	.56
117. I have lost respect for myself. (PMBS)	.67	.81	.42
123. I will not be able to tolerate my thoughts about the event, and I will fall apart. (PTCI experimental item)	.48	.83	.60
**Assimilation**			
7. I knew better than to do what I did. (TRGI)	.49	.55	.57
16. This event(s) could have been avoided. (created)	.63	.46	.62
34. I blame myself for what happened. (TRGI)	.69	.71	.80
37. I did something that went against my values. (TRGI)	.49	.64	.71
40. It would not have happened if I would have been paying attention. (created)	.79	.76	.61
46. I should have known better. (TRGI)	.82	.80	.73
61. I hold myself responsible for what happened. (TRGI)	.78	.80	.77
70. What I did was inconsistent with my beliefs. (TRGI)	.68	.74	.61
82. The event happened because I wasn’t careful enough. (TAQ)	.82	.79	.77
94. The event happened because of the way I acted. (PTCI)	.76	.80	.66
100. I could have prevented what happened to me. (TRGI)	.85	.79	.64
103. I blame myself for something I did, thought, or felt. (TRGI)	.66	.77	.77
106. I had some feelings that I should not have had. (TRGI)	.58	.62	.33
**Accommodation**			
11. I have made good and bad choices in life. (created)	.48	.51	.19
18. You never know when something terrible will happen. (PTCI experimental item)	.57	.52	.14
32. I will get upset if someone pushes me too far. (created)	.51	.42	.26
65. Life is sometimes a gamble. (WAS)	.53	.48	.76
68. Sometimes bad things happen for no good reason. (created)	.64	.56	.71
72. You can never know who will harm you. (PTCI)	.59	.46	.35
77. I did the best I could in an unpredictable situation. (created)	.44	.50	.08
83. Life is about surviving challenging events. (created)	.54	.53	.54
89. I have made some mistakes, but that does not make me a bad person. (created)	.54	.64	.36
92. The world has good and bad people in it. (created)	.63	.68	.45
104. Sometimes good people do bad things. (created)	.60	.63	.24
113. Overall, I am a good person despite some of my faults. (created)	.56	.64	.08
114. Danger is always present. (TAQ)	.59	.43	.51
131. One cannot always predict the outcome of a situation. (created)	.64	.60	.17
133. Sometimes bad things happen to good people. (created)	.73	.73	.50
**Optimism**			
2. I can trust my friends. (created)	.51	.45	.33
8. The good things that happen in this world far outnumber the bad. (WAS)	.55	.55	.50
12. Human nature is basically good. (WAS)	.77	.73	.47
19. By and large, good people get what they deserve in this world. (WAS)	.51	.49	.55
20. Some people can be trusted. (PMBS)	.54	.45	.14
33. I am very satisfied with the kind of person I am. (WAS)	.58	.56	.38
38. Most people are basically caring. (PMBS)	.74	.72	.70
57. People are basically kind and helpful. (WAS)	.83	.11^+^	.72
59. My emotions are typical of most people. (created)	.51	.60	.20
60. Other people can be genuinely loving toward me. (PMBS)	.58	.66	.52
67. People will experience good fortune if they themselves are good. (WAS)	.50	.50	.40
74. If you look closely enough, you will see that the world is full of goodness. (WAS)	.72	.76	.72
86. Most people are capable of good things. (created)	.59	.71	.59
95. There is more good than evil in this world. (WAS)	.70	.73	.71
129. I comfort myself very well when I’m upset. (PMBS)	.47	.47	.22
132. The world is a good place. (WAS)	.77	.78	.67

Phase 2: *N* = 815 non-clinical respondents. Phase 3: *N* = 651 non-clinical respondents who reported trauma. Phase 4: *N* = 73 clinical respondents. Item sources: *Posttraumatic Maladaptive Beliefs Scale* (PMBS; 6 items); *Posttraumatic Cognitions Inventory* (PTCI; 20 items); *Trauma Appraisal Questionnaire* (TAQ; 8 items); *Trauma-Related Guilt Inventory* (TRGI; 9 items); *World Assumptions Scale* (WAS; 10 items); and 15 newly created items.

Between 10% and 20% of data values were missing. A missing data rate of 15% to 20% is common in psychological studies [36]. These four factors explained a total of 49.83% of total response variation. Scale score means, standard deviations, and inter-correlations are presented in Table 3. Item endorsement on each scale suggested participants ‘disagreed’ to ‘somewhat disagreed’ with items assessing overaccommodation; were ambivalent about items that assessed assimilation, with average item ratings ranging between ‘somewhat disagree’ to ‘somewhat agree’; and generally agreed with items that assessed accommodation and optimism, with average item ratings between ‘somewhat agree’ and ‘agree.’ For each scale, internal consistency reliability was very good to excellent [37]. Overaccommodation was positively associated with assimilation and negatively associated with optimism; assimilation was positively associated with accommodation; and accommodation was positively associated with optimism (Table 3).

**Table 3:** Descriptive statistics, inter-scale correlations, and internal consistency reliability estimates of the TRCS subscales in the Phase 2 (*N* = 815) and Phase 3 (*n* = 651) non-clinical samples.

	(1)	(2)	(3)	(4)
Correlations				
(1) Overaccommodation	--	.68***	-.07	-.16***
(2) Assimilation	.68***	--	.14***	.03
(3) Accommodation	-.03	.11*	--	.49***
(4) Optimism	-.14**	.05	.37***	--
Means (SD)				
Phase 2	2.39 (1.14)	3.05 (1.21)	4.50 (0.78)	4.17 (0.88)
Phase 3	2.35 (1.14)	3.04 (1.19)	4.51 (0.76)	4.13 (0.82)
Internal Consistency Reliability				
Phase 2	.97	.94	.89	.93
Phase 3	.97	.93	.87	.84

Note.

***p < .001

**p < .01

*p < .05.

Correlation entries above the diagonal are Phase 2 inter-scale Pearson *r* correlations; correlations below the diagonal are Phase 3 inter-scale Pearson *r* correlations. Item response options: 1 (*strongly disagree*), 2 (*disagree*), 3 (*somewhat disagree*), 4 (*somewhat agree*), 5 (*agree*), 6 (*strongly agree*).

## Phase 3: Non-clinical sample #2 confirmatory factor analysis and validity assessment

### Phase 3 method

#### Phase 3 participants and recruitment procedure

The Phase 3 sample initially included 998 community individuals recruited through Amazon’s MTurk in 2014. Research has found MTurk samples are more attentive to instructions than traditional subject pool samples [38]. Participants age 18 and above provided electronic consent to complete the measures online. Following the completion of the study, participants were thanked for their participation and provided a debriefing form.

The majority of participants identified as male (51.2%) and identified as Caucasian/White (57.4%); 14.9% identified as African American, 18.9% identified as Asian, 1% identified as American Indian/Alaskan Native, 3.3% identified as biracial, and 2.2% reported ’unknown’; 11.5% identified their ethnicity as Hispanic or Latino. About a quarter of participants had a high school degree/GED (24.4%), 0.9% reported having some high school, 39.4% reported having some college/vocational training, 22.3% reported a college degree, 1.3% reported some graduate training, and 11.7% reported having a graduate degree. The majority were employed full-time (31.7%) or part-time (27.8%). Most participants had experienced a potentially traumatic event (see Table 1).

For psychometric analyses, the sample was restricted to those who reported experiencing at least one interpersonal trauma (i.e., an event in which another human being inflicts physical or psychological injury on another human being; *n* = 651). Interpersonal traumas were selected based upon TLEQ responses using the same procedure as Frazier and colleagues [39], except for abortion, which was not conceptualized in this study as an interpersonal trauma. Participants selected for interpersonal trauma, compared to those who were dropped from analyses, reported significantly more posttraumatic stress on the PTSD Checklist-Civilian Version (PCL-C; described below) [40], *t*(994) = 8.34, *p* < .001. Using the most stringent cut-point score of 50, 25.8% would be considered PTSD positive in the interpersonal trauma sub-sample; whereas, 12.7% in the non-trauma sub-sample would be considered PTSD positive.

#### Phase 3 measures

*Trauma cognitions*. The reduced 69-item TRCS from the previous phase was used.

*Trauma history*. The TLEQ was used to assess potential traumatic experiences.

*PTSD symptoms*. The PTSD Checklist-Civilian Version (PCL-C) [40] was used to assess PTSD symptoms. The PCL-C is a 17-item self-report scale for posttraumatic stress based on *DSM-IV* criteria. Items are rated from 1 (*Not at All*) to 5 (*Extremely*). A total severity score is obtained by summing scores from each of the 17 items. The PCL-C has demonstrated high internal consistency ranging from .92 to .96 in community samples, and has shown convergent validity with other measures of posttraumatic stress and discriminant validity with measures of separate psychological constructs [41]. Internal consistency in the subsample selected for interpersonal trauma was *α* = .95.

*Depressive symptoms*. The Beck Depression Inventory-II **(**BDI-II) [42] is a 21-item self-report measure for assessing the severity of depression. Items are scored from 0 to 3 with different anchors for each item (e.g., 1 = *I do not feel sad* to 3 = *I am so sad or unhappy that I can’t stand it*), with higher scores indicating more severe depressive symptoms. The average test-retest reliability is *r* = .93 and the average internal consistency is *α* = .92 [42]. In the Phase 3 sample, internal consistency was *α* = .94.

*Resilience*. The Brief Resilience Scale [43] is a 6-item measure that operationalizes resilience as bouncing back from stress (“It does not take long to recover from a stressful event”). Items are rated from 1 (*Strongly disagree*) to 5 (*Strongly agree*). Three items are reverse scored, and all items summed, with higher scores indicating greater resiliency. Internal consistency for this scale is adequate, ranging from .80 to .91 across four samples [43]. In the Phase 3 sample, internal consistency was *α* = .85.

*Personality*. The Big Five Inventory [44] consists of 44 short-phrase items, rated on a five-point scale from 1 (*Disagree Strongly*) to 5 (*Agree Strongly*), that represent the core traits that define the Big Five personality domains of Extraversion (“Is outgoing, sociable”), Neuroticism (“Worries a lot”), Conscientiousness (“Does a thorough job”), Agreeableness (“Has a forgiving nature”), and Openness to Experience (“Is ingenious, a deep thinker”). Internal consistency in this sample ranged from .74 (Agreeableness) to .86 (Neuroticism).

*Trait anxiety*. The State-Trait Anxiety Inventory [45] was used to assess trait-anxiety, a relatively stable individual difference in anxiety proneness and the tendency to perceive stressful situations as dangerous or threatening. Trait anxiety was measured with 10 items (“I feel nervous and restless”) on a 4-point scale ranging from 1 (*Almost Never*) to 4 (*Almost Always*). Internal consistency across different samples ranged from .89 to .91. Internal consistency in this sample was (*α* = .91).

*Trauma beliefs*. The TABS [26] is an 84-item self-report assessment of cognitive schemas (beliefs about oneself and about others). The TABS measures beliefs related to five need areas that are sensitive to the effects of traumatic experiences: safety, trust, esteem, intimacy, and control. Within each need area, separate sets of items tap into beliefs about oneself and beliefs about others. Items are rated from 1 (*Disagree Strongly*) to 6 (*Agree Strongly*), with higher scores indicating more negative beliefs about oneself and others. Internal consistency (.67 to .87) and test-retest reliabilities (.60 to .79) for the TABS subscales are adequate [26]. Internal consistency in this sample ranged from .85 (Intimacy) to .92 (Control).

#### Phase 3 data analysis plan

A confirmatory factor analysis (CFA) model was fit using LISREL 8.72 as a confirmatory test of the 69-item, four-factor TRCS measurement model empirically identified by the Phase 2 EFA model. Model fit was assessed by the Satorra-Bentler scaled chi-square (*χ*^*2*^_SB_) [46], the root mean squared error of approximation (RMSEA) [47], and the comparative fit index (CFI) [48]. RMSEA values ≤.06 and CFI values ≥ .95 are thought to suggest approximate model fit [49]. Across 69 items, 3.3% of data values were missing. To accommodate missing data the CFA model was fit to multiply imputed data and the asymptotic covariance matrix of item variances and covariances was estimated via the bootstrap. Following CFA modeling, scale scores were calculated for each factor. Inter-scale correlations and Cronbach alphas were estimated.

### Phase 3 results

#### Phase 3 confirmatory factor analysis

The 4-factor, 69-item CFA model fit well: Satorra-Bentler χ^2^(2271) = 7394.72, *p* < .0001; *RMSEA* = 0.059; *CFI* = 0.967. See Table 2 for factor loadings. The four factors demonstrated high internal consistency reliability in the Phase 3 sample of non-clinical participants selected for interpersonal trauma exposure: Overaccommodation (*α* = .97), Assimilation (*α* = .93), Accommodation (*α* = .87), Optimism (*α* = .90). Scale score means, standard deviations, and inter-correlations are presented in Table 3. In this non-clinical sample selected for interpersonal trauma, it was found that item endorsement on each scale suggested participants ‘disagreed’ to ‘somewhat disagreed’ with items assessing overaccommodation; were ambivalent about items assessing assimilation, with average item ratings ranging between ‘somewhat disagree’ to somewhat agree’; and generally agreed with items assessing accommodation and optimism, with average items ratings between ‘somewhat agree’ and ‘agree.’ Again, similar to Phase 2 results, overaccommodation was highly positively associated with assimilation and negatively associated with optimism; assimilation was positively associated with accommodation; and accommodation was moderately positively associated with optimism.

#### Phase 3 criterion-related validity

*Known groups validity*. Not surprisingly, participants from the Phase 3 sample who endorsed experiencing interpersonal trauma(s), compared to those who did not, scored significantly higher on Overaccommodation (*t*[993] = 4.24, *p* < .001), Assimilation (*t*[990] = 4.37, *p* < .001), and Accommodation (*t*[993] = 4.71, *p* < .001), although there was no significant difference on Optimism (*t*[993] = 0.99, *p* = .337). Additionally, increased endorsement of interpersonal traumas was associated with more overaccommodation, assimilation, accommodation, and less optimism (Table 4; *p*s > .05).

**Table 4:** Evidence of criterion-related validity: Phase 3 sample (N = 651).

	Overaccommodation	Assimilation	Accommodation	Optimism
*Known Groups*				
TLEQ Total Interpersonal Trauma	.25***	.16***	.15*	-.09***
*Convergent and Discriminant*				
TABS–Safety	.80***	.57***	-.09*	-.23***
TABS–Trust	.67***	.45***	-.12**	-.47***
TABS–Esteem	.77***	.52***	-.11**	-.45***
TABS–Intimacy	.72***	.50***	-.05	-.36***
TABS—Control	.81***	.61***	.06	-.13**
STAI–Trait Anxiety	.49***	.32***	-.01	-.46***
BFI–Neuroticism	.33***	.20***	.08*	-.39***
BFI–Extraversion	-.13**	.00	.02	.38***
BFI–Agreeableness	-.36***	-.18***	.12**	.33***
BFI–Conscientiousness	-.33***	-.19***	.19***	.24***
BFI—Openness	-.07	.00	.25***	.21***
*Concurrent*				
BRS–Resilience	-.51***	-.34***	.01	.36***
BDI-II–Depression Symptoms	.61***	.43***	.06	-.29***
PCL–PTSD Symptoms	.68***	.51***	.09*	-.11**

Note.

***p < .001

**p < .01

*p < .05.

TLEQ = Traumatic Life Events Questionnaire. TABS = Trauma and Attachment Beliefs Scale. STAI = State-Trait Anxiety Inventory. BFI = Big Five Inventory. BRS = Brief Resilience Scale. BDI = Beck Depression Inventory. PCL = PTSD Checklist.

*Convergent and discriminant validity*. See Table 4 for bivariate correlations between the four TRCS subscales and both trauma beliefs and personality factors.

*Concurrent validity*. The TRCS subscales correlated highly and in expected directions with measures of resilience and psychopathology, including depression and posttraumatic stress (see Table 4). A multiple linear regression analysis modeling resilience, with TRCS positive subscales entered simultaneously, found that optimism (standardized *β* = .43, *p* < .001) and accommodation (*β* = -.14, *p* < .001) were unique predictors of resilience, though in opposite directions (*Adjusted R*^*2*^ = .16, *F* [2, 616] = 57.51, *p* < .001). A multiple linear regression analysis modeling posttraumatic stress symptom severity, with TRCS negative subscales entered simultaneously, found that both assimilation (*β* = .09, *p* = .019) and overaccommodation (*β* = .62; *p* < .001) were unique predictors of posttraumatic stress (*Adjusted R*^*2*^ = .47, *F* [2, 649] = 287.49, *p* < .001). A multiple linear regression analysis modeling depression symptom severity, with TRCS negative subscales entered simultaneously, found only overaccommodation (*β* = .59, *p* < .001) and not assimilation (*β* = .03; *p* = .473) was a unique predictor of depression (*Adjusted R*^*2*^ = .37, *F* [2, 648] = 193.87, *p* < .001).

*Incremental validity*. To examine whether there was a relation between the TRCS negative subscales and posttraumatic stress symptom severity when variation in trauma- and attachment-related beliefs were controlled, we fit two linear regression models including the five trauma- and attachment-related beliefs from the TABS (trust, safety, control, esteem, intimacy) entered on step 1 and either Overaccommodation (*Adjusted R*^*2*^ = .50; *F* [6, 643] = 108.54, *p* < .001) or Assimilation (*Adjusted R*^*2*^ = .46; *F* [6, 643] = 90.44, *p* < .001) entered on step 2 in two different models. Overaccommodation (Δ*R*^*2*^ = .07, *β* = .50, *p* < .001) and Assimilation (Δ*R*^*2*^ = .02, *β* = .20, *p* < .001) remained unique predictors of posttraumatic stress after controlling for trauma- and attachment-related beliefs.

## Phase 4: Clinical sample confirmatory factor analysis and validity assessment

### Phase 4 method

#### Phase 4 participants and recruitment procedure

Participants included 73 acute victims of crime who were presenting for weekly trauma-focused therapy from 2015 to 2016 with one of 17 clinicians at a community mental health clinic in a large metropolitan area on the U.S. West Coast. Clinicians at the community mental health clinic approached potential clients during an intake evaluation about participating in the study, and those who expressed interest in participating met with a research assistant to learn more about the study. After providing written informed consent, participants were given a questionnaire packet that took approximately 45 minutes to complete. Demographic data, trauma history, and psychiatric diagnoses assessed at intake were extracted from clinical records. Following the completion of the packet of questionnaires, participants were thanked for their time and debriefed. They were provided a payment of $20 for their time.

The average age of participants was 41.28 (*SD* = 13.80), ranging from 21 to 87. The majority of participants identified as female (60.3%). Participants were racially diverse: 33.2% identified as Hispanic, 24.7% identified as Caucasian/White, 13.7% identified as African American/Black, 9.6% identified as Asian/Pacific Islander, 1.4% identified as biracial, 15.1% specified ’Other’ as their race, and 1.4% declined to provide a response. The majority of participants identified as heterosexual (58.9%); 13.7% identified as homosexual, 5.5% identified as bisexual, 21.9% either did not provide or declined to provide their sexual orientation.

#### Phase 4 measures and summary statistics

*Trauma cognitions*. In Phase 4, the reduced 69-item TRCS that resulted from the Phase 2 EFA and that was confirmed in the Phase 3 CFA was administered.

*Trauma history*. Trauma history was assessed in 63 participants with the Trauma History Screen (THS) [50] during an intake evaluation at the community mental health clinic before clients enrolled in trauma services at the clinic. The THS is a 14-item self-report measure that assesses exposure to a variety of traumatic events, 13 specific types of events (e.g., car accident, sexual assault, physical assault, natural disaster, etc.) and one “other” event. During the intake interview, for each event, participants were asked to indicate whether they experienced the event (“yes” or “no”). The average number of types of trauma experienced, as assessed by the THS, was 4.81 (*SD* = 2.93) and ranged up to 12 discrete traumas (Table 5).

**Table 5:** Exposure to lifetime potential trauma in Phase 4 clinical sample (*N* = 73).

Trauma Type	Valid %
1. A really bad car, boat, train, or airplane accident	29.8
2. A really bad accident at work or home	23.2
3. A hurricane, flood, earthquake, tornado, or fire	33.9
4. Hit or kicked hard enough to injure–as a child	45.9
5. Hit or kicked hard enough to injure–as an adult	63.8
6. Forced or made to have sexual contact–as a child	44.1
7. Forced or made to have sexual contact–as an adult	50.0
8. Attacked with a gun, knife, or weapon	--
9. During military service–seeing something horrible or being badly scared	2.2
10. Sudden death of close family or friend	71.9
11. Seeing someone die suddenly or get badly hurt or killed	43.6
12. Some other sudden event that made you feel very scared, helpless, or horrified	49.0
13. Sudden move or loss of home and possessions	38.5
14. Suddenly being abandoned by a spouse, partner, parent, or family	43.4

*Note*. Item 8 was not included in the assessment. Valid percentages are used for each item because not all clinicians assessed trauma history during the intake; there is missing data for each item, ranging between 12 (Item 4) and 27 (Item 9) participants with missing data.

To be eligible for trauma services at the clinic, the client had to be a victim of a crime within the last 3 years, or a survivor of torture in another country, or a family member of a homicide victim. The index trauma that precipitated mental health treatment at the clinic was identified for 64 participants during the intake evaluation. Most participants identified sexual assault (32.9%), followed by physical assault (26%); 9.6% were family members of homicide victims, 5.5% were refugees or victims of torture in their home country, 4.1% were victims of vehicular assault, 2.7% were victims of a stabbing, 2.7% were victims of attempted murder, 1.4% were victims of a shooting, and 2.7% of participants reported another type of crime as their index trauma.

*Psychiatric diagnoses*. Psychiatric diagnoses were assessed for 63 participants during an intake evaluation before clients enrolled in trauma services at the community mental health clinic by clinicians who conducted a clinical interview and utilized symptom checklists to identify psychiatric symptoms. After the intake, clinicians met weekly for an intake meeting to solidify psychiatric diagnoses made during the intake. Almost all of the sample (92.1%) met criteria for PTSD or another Trauma-and-Stressor-Related Disorder as defined by the *DSM-5* [12]. The average number of diagnoses was 2.11 (*SD* = 1.44), ranging from zero (two participants did not meet criteria for any disorder) to eight (in the case of multiple substance use disorders), with a Depressive Disorder being the most common co-morbid diagnosis (39.7%). Other diagnoses included a Substance Use Disorder (19.1%), Anxiety Disorder (16.4%), Bipolar Disorder (4.8%), Psychotic Disorder (3.2%), Eating Disorder (3.2%), or Gender Dysphoria (1.6%).

*PTSD symptoms*. The PTSD Checklist-Civilian Version (PCL-C) [40] and the PTSD Checklist for *DSM-5* (PCL-5) [51] were both used to assess PTSD symptoms. The PCL-5 is a 20-item self-report scale for posttraumatic stress based on *DSM-5* criteria. Items are rated from 0 (*Not at All*) to 4 (*Extremely*). A total severity score is obtained by summing scores from each of the 20 items. Internal consistency for the PCL-5 has ranged from .91 (military service member sample seeking treatment) [52] to .94 (college sample) [53]. Part way through data collection, the clinic switched from using the PCL-C to the PCL-5 to assess posttraumatic stress as the new PCL version became available after the release of the *DSM-5* [12]. Researchers at the clinic created a crosswalk between the two versions (i.e., a scoring rubric was created to produce scores that were equivalent across both versions of the measure). The PCL total is used as the PTSD severity score in this Phase. The PCL was administered prior to initiating trauma-focused therapy (baseline), as well as the beginning of sessions 8 and 16. Internal consistency for the PCL total in this sample ranged from .89 (baseline) to .93 (session 16).

*Depressive symptoms*. The Patient Health Questionnaire-9 [54] is a 9-item self-report assessment that can be used as a continuous measure of depressive symptoms. Items ask participants to rate if they have been bothered by each symptom (e.g., “Little pleasure or interest in doing things”) in the past 2 weeks on a scale from 0 (*not at all*) to 3 (*nearly every day*). A total severity score is obtained by summing the scores from each item. Internal consistency has ranged from .86 to .92 in several different samples [55]. The PHQ-9 was administered prior to initiating trauma-focused therapy (baseline), as well as the beginning of sessions 8 and 16. Internal consistency in this sample ranged from .79 (baseline) to .89 (therapy session 16).

*Resilience*. The Brief Resilience Scale [43] was used to assess resilience. In the Phase 4 sample, internal consistency was α = .84.

*Personality*. The Big Five Inventory [44] assessed the Big Five personality domains. Internal consistency ranged from .70 (Agreeableness) to .83 (Neuroticism).

*Trait anxiety*. The State-Trait Anxiety Inventory [45] was used to assess trait-anxiety. Internal consistency in this sample was (α = .91).

*Trauma beliefs*. The TABS [26] assessed beliefs related to five need areas that are sensitive to the effects of traumatic experiences- safety, trust, esteem, intimacy, and control. Internal consistency in this sample ranged from .78 (Control) to .83 (Trust).

#### Phase 4 data analysis plan

Again, a four-factor confirmatory factor analysis (CFA) model was fit to the 69-item TRCS data. Across 69 items, 3.3% of data values were missing. Across 69 items, 0.54% of data values were missing. To accommodate missing data the CFA model was fit to multiply imputed data and the asymptotic covariance matrix of item variances and covariances was estimated via the bootstrap. Following CFA modeling, scale scores were calculated for each factor, and reliability and validity assessments conducted.

### Phase 4 results

#### Phase 4 confirmatory factor analysis

The TRCS 69-item, 4-factor CFA model was fit to clinical sample data: Satorra-Bentler χ^2^_SB_(2271) = 2858.91, *p* < .001; *RMSEA* = 0.060; *CFI* = 0.916. Several factor loadings had low values (Table 2) and given the relatively low CFI value, we next fit a modified model that dropped the 18 items with factor loadings <0.409. The resulting 51-item, 4-factor model had improved fit: Satorra-Bentler χ^2^(1218) = 1523.08, *p* < .001; *RMSEA* = 0.059; *CFI* = 0.948.

Item endorsement on each scale suggested participants ‘disagreed’ to ‘somewhat disagreed’ with items assessing overaccommodation; were ambivalent about items that assessed assimilation, with average item ratings ranging between ‘somewhat disagree’ to ‘somewhat agree’; and generally agreed with items that assessed accommodation and optimism, as average item ratings were between ‘somewhat agree’ to ‘agree’ (see Table 6). Internal consistency reliability ranged from .62 (accommodation) to .92 (overaccommodation). Overaccommodation was positively and negatively associated with assimilation and optimism, respectively. However, accommodation was not significantly associated with any subscale.

**Table 6:** Descriptive statistics, inter-scale correlations, and internal consistency reliability estimates of the 69-item TRCS subscales in Phase 4 clinical sample (*N* = 73).

	(2)	(2)	(3)	(4)
Correlations				
(1) Overaccommodation	--			
(2) Assimilation	.46***	--		
(3) Accommodation	.22	-.09	--	.
(4)Optimism	-.40***	-.15	-.18	--
Means (SD)				
Phase 4	2.68 (0.79)	3.14 (1.09)	4.87 (0.44)	4.18 (0.65)
Internal Consistency Reliability				
Phase 4	.92	.91	.62	.83

Note.

***p < .001

**p < .01

*p < .05.

Item response options: 1 (strongly disagree), 2 (disagree), 3 (somewhat disagree), 4 (somewhat agree), 5 (agree), 6 (strongly agree).

#### Phase 4 criterion-related validity

*Convergent and discriminant validity*. See Table 7 for bivariate correlations between the four TRCS subscales and both trauma beliefs and personality factors.

**Table 7:** Evidence of criterion-related validity: Phase 4 sample (*N* = 73).

	Overaccommodation	Assimilation	Accommodation	Optimism
*Convergent and Discriminant*				
TABS–Safety	.47***	.22	-.23	-.40**
TABS–Trust	.53***	.26*	.06	-.53***
TABS–Esteem	.51***	.33**	.07	-.55***
TABS–Intimacy	.58***	.40**	.12	-.43***
TABS—Control	.49***	.27*	.11	-.40**
STAI–Trait Anxiety	.75***	.40**	.25*	-.62***
BFI–Neuroticism	.60***	.23	.37**	-.60***
BFI–Extraversion	-.25**	.02	-.03	08
BFI–Agreeableness	-.43***	-.12	-.06	.24*
BFI–Conscientiousness	-.38**	-.34**	-.05	.15
BFI—Openness	-.18	-.09	.10	.02
*Concurrent*				
BRS–Resilience	-.49***	-.26*	-.23	.35**
BDI-II–Depression Symptoms	.53***	.30	.22	-.28*
PCL–PTSD Symptoms	.39**	.10	.33**	-.25*

Note.

***p < .001

**p < .01

*p < .05.

TLEQ = Traumatic Life Events Questionnaire. TABS = Trauma and Attachment Beliefs Scale. STAI = State-Trait Anxiety Inventory. BFI = Big Five Inventory. BRS = Brief Resilience Scale. BDI = Beck Depression Inventory. PCL = PTSD Checklist.

*Concurrent validity*. The TRCS subscales correlated in expected directions with measures of resilience, depression, and posttraumatic stress (see Table 7). A multiple linear regression analysis modeling resilience, with TRCS positive subscales entered simultaneously, found only optimism (*β* = .32, *p* = .008) and not accommodation (*β* = -.18, *p* = .129) was a unique predictor of resilience (*Adjusted R*^*2*^ = .13, *F* [2, 67] = 5.88, *p* = .005). A multiple linear regression analysis modeling posttraumatic stress symptom severity, with TRCS negative subscales entered simultaneously, found only overaccommodation (*β* = .44, *p* = .001) and not assimilation (*β* = -.11; *p* = .394) was a unique predictor of posttraumatic stress (*Adjusted R*^*2*^ = .14, *F* [2, 72] = 6.78, *p* = .002). A multiple linear regression analysis modeling depression symptom severity, with TRCS negative subscales entered simultaneously, found only overaccommodation (*β* = .50, *p* < .001) and not assimilation (*β* = .07; *p* = .562) was a unique predictor of depression (*Adjusted R*^*2*^ = .26, *F* [2, 72] = 13.55, *p* < .001).

*Incremental validity*. To examine whether there was a relation between the TRCS negative subscales and posttraumatic stress symptom severity when variation in trauma- and attachment-related beliefs were controlled, we fit two linear regression models including the five trauma- and attachment-related beliefs from the TABS entered on step 1 and either Overaccommodation (*Adjusted R*^*2*^ = .10; *F* [6, 68] = 1.07, *p* = .044) or Assimilation (*Adjusted R*^*2*^ = .00; *F* [6, 68] = 0.95, *p* = .466) entered on step 2 in two different models. Overaccommodation (Δ*R*^*2*^ = .11, *β* = .41, *p* = .006) but not Assimilation (Δ*R*^*2*^ = .01, *β* = .09, *p* = .516) was a unique predictor of posttraumatic stress after controlling for trauma- and attachment-related beliefs.

*Predictive validity*. The PCL and the PHQ9 were assessed longitudinally. For each outcome, we fit five linear mixed effect longitudinal models. The first model included the 3-category assessment time indicator as the only *X* variable (baseline/pre-therapy, week 8, and week 16). This base model estimated the longitudinal effect of therapy on the corresponding outcome. Subsequently, four augmented models were fit; each included one TRCS scale as well as the interaction between the TRCS scale and the assessment time indicator. Non-significant interaction effects were dropped and the augmented model refit. Augmented models with no interaction term estimated the time-averaged effect of the modeled TRCS scale on the corresponding outcome. Models including an interaction term estimated time-specific effects of the modeled TRCS scale on the outcome. Each model included random effects for clinicians and patients and assumed data were missing at random (MAR), conditional on modeled variables.

Both the PCL and PHQ9 were completed by 73 (100%), 64 (87.7%), and 47 (64.4%) clients at the three assessments, respectively. The PCL means equaled 47.7, 27.7, and 22.8 at the three assessments. Base model tests of the baseline PCL mean versus the 8- and 16-week means were significant (both *p* < .001) and the 8- and 16-week means significantly differed (*p* = .036); this pattern also was observed in each augmented model. The interaction term was dropped from each augmented PCL model (*p*-values ranged from .217 to .826). Linear regression parameter estimates for the time-averaged effect of each TRCS scale on PCL scores follow: overaccommodation, *b* = 9.61, *p* < .001; assimilation, *b* = 0.03, *p* = .982; accommodation, *b* = 13.16, *p* < .001; and optimism, *b* = −8.72, *p* < .001. For example, a one-point increase on the TRCS overaccommodation scale corresponded to an expected 9.61-point increase on the PCL.

The PHQ9 means equaled 14.9, 7.4, and 7.6 at the three assessments. Base model tests of the baseline PHQ9 mean versus the 8- and 16-week means were significant (both *p* < .001), but the 8- and 16-week means did not significantly differ (*p* = .867); this pattern held in each augmented model. The interaction effect was dropped from three of the augmented PHQ9 models (*p*-values ranged from .667 to .984) and the linear regression parameter estimates for the corresponding time-averaged effects follow: overaccommodation, *b* = 3.90, *p* < .001; accommodation, *b* = 3.76, *p* = .004; and optimism, *b* = −8.72, *p* < .001. A significant interaction effect between assimilation and assessment time (*p* = .048) resulted in time-specific effects of assimilation on PHQ9: pre-therapy, *b* = 1.80, *p* = .008; 8 weeks, *b* = −0.08, *p* = .909; and 16 weeks *b* = 0.22, *p* = .785). The pre-therapy effect significantly differed from the 8-week effect (*p* = .021) and marginally differed from the 16-week effect (*p* = .071). Thus, assimilation was significantly related to pre-therapy PHQ9 scores, but that relationship no longer held by the 8-week therapy session.

## Discussion

The aim of this research was to develop a measure that assesses cognitions consistent with cognitive processes of assimilation, accommodation, and overaccommodation. The Cognitive Theory of PTSD [56] asserts PTSD develops and persists in the aftermath of traumatic events that alter or reinforce negative cognitions about oneself, people, and the world. Cognitive Processing Therapy (CPT) [7] follows from this theory, arguing that PTSD symptomatology endures among individuals who assimilate (e.g., “I should not have gotten drunk that night”) or over-accommodate (e.g., “All men are evil”) the traumatic event into their core cognitive schemas about the world. A randomized clinical trial to dismantle the active components of CPT [57] demonstrated evidence for belief change as a mechanism of treatment success, highlighting the importance of identifying and eventually accommodating trauma-related cognitions into a schema system that recognizes the world as imperfect, but not wholly malevolent (e.g., “Sometimes people do bad things”).

Despite evidence that post-trauma cognitions consistent with maladaptive information-processing play a role in PTSD onset, maintenance, and recovery, post trauma cognition measures used in research, treatment planning, and symptom monitoring typically reflect cognitions associated with just one cognitive process. Few measures incorporate items reflective of accommodated cognitions, which are items that would likely be most indicative of treatment-related success. The present study used two non-clinical samples and one clinical sample to develop a measure of cognitions consistent with all three cognitive processes, with an unanticipated fourth factor reflecting optimism emerging from analyses. Though preliminary, the measure has potential to impact the trauma field by providing empirical support for cognitive theories of PTSD, revealing the stability of post-trauma cognitions with and without intervention, and identifying idiosyncratic cognitions that may be targeted by clinicians providing trauma-focused treatment or cognitions that predict symptom trajectory.

Across the three samples, participants generally disagreed with items on overaccommodation, scored in the mid-range for assimilation items, and agreed with cognitions associated with accommodation and optimism. This may be anticipated in regard to Phase 3 results, as the majority of the participants did not endorse clinical levels of PTSD symptomatology despite exposure to interpersonal trauma. It is possible the overaccommodated and assimilated cognitions are more specific to PTSD development than trauma exposure in the absence of PTSD symptoms. However, the same pattern of results was found in the clinical sample. This may indicate that clinical samples hold idiosyncratic trauma cognitions that may not reflect average scores on the subscale. With this in mind, clinicians may use this measure as a tool to assess which idiosyncratic cognitions are most relevant to that individual and track those specific cognitions over therapy. Outcome research using the TRCS will shed light on whether the average scores found in this study replicate in subsequent clinical samples, as well as determine the clinical utility of the measure.

Contrary to expectations, a fourth factor emerged with items that reflect post-trauma optimism. Although unanticipated, the optimism domain makes theoretical sense, as some individuals may endorse cognitions about themselves and their recovery that are more purely positive in nature rather than the more neutral cognitions associated with accommodation or the more maladaptive cognitions associated with assimilation or over-accommodation. Though the optimistic cognitions factor emerged across all three subsamples, the extent to which the optimism factor has utility in research or clinical contexts remains an empirical question.

Several limitations to the current study must be noted. Phases 2 and 3 used Mechanical Turk to collect data, which may have increased selection effects [58]. As is common in measure development, the initial 4-factor CFA model fit to the clinical sample did not fit the data adequately, and thus the CFA model was empirically modified by dropping some items; yet, the number of factors and the groupings of remaining items within factors were unchanged. Therefore, the TRCS measurement model within clinical populations should be considered provisional until tested in subsequent clinical samples with a larger number of participants. In addition, the present study did not assess for several factors that have been implicated in post-trauma cognitions and reactions. For example, time elapsed since an index traumatic event was not assessed. Survivors’ cognitions regarding an event may change naturally over time, or be altered with formal or informal intervention, the latter of which was also not assessed. Future research that considers how time elapsed since participants’ index traumatic event, and previous exposure to intervention, impact cognitions assessed by the TRCS is needed. Further, in the non-clinical trauma sample in Phase 3 of the present study, the extent to which the potentially traumatic events assessed by the TLEQ resulted in traumatization (and therefore were truly traumatic) was not assessed. However, use of a cut-off score to examine the prevalence of probable PTSD in interpersonal trauma (IPT) survivors (versus those without such exposure) revealed nearly 25% of IPT survivors met cut-off for probable PTSD using a conservative cut point. As such, it is reasonable to assume that a sizable minority of the IPT survivors found their index event to be traumatizing. This limitation was largely rectified in the last phase, as 91% qualified for a diagnosis of PTSD and all participants were seeking trauma-focused treatment. However, future studies using the TRCS would benefit from collecting additional samples of PTSD-positive participants, as well as assessing associations between subscales of the TRCS and symptoms of other disorders frequently observed in trauma survivors (i.e., depression, anxiety, substance use). Further, additional information regarding participants’ trauma history is important to collect to ensure those indicating potential exposure have experienced an event that would reasonably impact trauma-related cognitions. In addition, items reflecting more general responses (i.e., “I’m not safe”) should be investigated further to determine whether these are trauma-specific or specific to post-trauma mental health outcomes.

Validity analyses in Phase 4 revealed preliminary evidence of concurrent validity, incremental validity, and predictive validity. Yet, replication is needed to further establish the validity of the scale. Additionally, it is important to note the internal consistency of the accommodation scale in Phase 4 was α = .62, which was collected at intake for trauma-focused treatment. It is likely accommodated beliefs are less consistent, as survivors of trauma may have idiosyncratic cognitions based on the nature of their trauma. For instance, it may be that an individual who was assaulted by a stranger holds negative cognitions related to safety, but may have fewer negative cognitions related to self-blame.

The instructions given on the TRCS may have also impacted study results. The instructions request that respondents consider the items in relationship to “a traumatic experience” without explicit definition of what a traumatic event entails. Inclusion of a definition of trauma or reference to a specific type of trauma may better orient participants to a life event that is truly traumatic. At present, it cannot be determined whether participants across the phases of the present study were considering a traumatic event that would fall within the *DSM-5* criteria for trauma when responding to TRCS items, or whether they were considering a life event that fails to achieve this criterion. Further, the instructions request that participants consider the degree to which items represent their thinking and agreement with the statements. It is possible for participants to have recurrent cognitions with which they do not agree. As such, future research that incorporates changes to the TRCS instructions or response scale may improve the interpretability and utility of findings.

The aim of the current study was to develop a comprehensive measure of post-trauma cognitions. The resulting scale may have utility in research and clinical contexts; however, the length of the scale may be a barrier to its use. Future research to identify items with the greatest utility that maintain scale validity is needed. Further, test-retest reliability was not assessed in the current study and should be an area for targeted investigation in future research. Information on the stability of posttraumatic cognitions with and without intervention would represent an important contribution to the traumatology field. As data from the first three phases of this study were collected prior to the release of the PCL-5 [51], additional research is needed to determine whether the scale maintains its association with PTSD symptoms using DSM-5 criteria in non-clinical samples. The PCL-5 was used in Phase 4 with the clinical sample.

A strength of this study was demonstration that the four-factor structure of the TRCS was replicated across different samples, including university students, community participants, and a treatment-seeking clinical sample. Despite study limitations, a provisional instrument of cognitions associated with varied post trauma cognitive processes was developed across several phases using both non-clinical and clinical samples. Incorporation of the TRCS in future survey research and clinical trials will determine with greater certainty its valid and reliable assessment of the four post trauma cognition factors identified in the current study, and of particular interest, whether changes in TRCS scores are associated with enhanced treatment-related outcomes. At the present time, trauma clinicians may consider employing TRCS for descriptive purposes as part of treatment planning and monitoring.

## Supporting information

S1 Appendix Items dropped from Phase 2 exploratory factor analysis. (DOCX)

S2 Appendix Trauma-Related Cognitions Scales. (DOCX)
