# Supplementary material for: Development and validation of the Trauma-Related Cognitions Scale
Source: PLoS One. 2021 Apr 15;16(4):e0250221. doi: 10.1371/journal.pone.0250221 (PMC8049256; doi:10.1371/journal.pone.0250221)
Supplement: S1 Appendix — (DOCX) [file pone.0250221.s001.docx]

**S1 Appendix. Items Dropped from Phase 2 Exploratory Factor Analysis.**

____________________________________________________________________________________________________________

Item Dropped from Exploratory Factor Analysis Hypothesized Factor Source

_________________________________________________________________________________________________________

01. There was probably no way I could have prevented what happened Accommodation Created

02. I make good decisions in most situations Accommodation Created

03. I can trust my own judgment even though it is not perfect Accommodation Created

04. I am not a weak person even though I have some weaknesses Accommodation Created

05. I typically handle my emotions well Accommodation Created

06. I can be lovable Accommodation Created

07. Some people are more trustworthy than others Accommodation Created

08. I have some control over what happens to me Accommodation Created

09. I have some control over my reactions to events Accommodation Created

10. I got through this so I can get through anything Accommodation Created

11. There are some people I can get close to Accommodation Created

12. There are some people that I can rely on Accommodation Created

13. I accept that this event happened to me Accommodation Created

14. I am realistic in my expectations of others Accommodation Created

15. Most people think of me as a good person Accommodation Created

16. I forgive myself for mistakes Accommodation Created

17. Bad events can teach us important, valuable lessons Accommodation Created

18. Certain situations should be avoided because they are dangerous Accommodation Created

19. I have a few people that I can confide in Accommodation Created

20. Life is what you make of it Accommodation Created

21. My life experiences have made me stronger Accommodation Created

22. Bad events are distributed to people at random Accommodation WAS

23. The course of our lives is mostly determined by chance* Accommodation WAS

24. Life can be too full of uncertainties that are determined by chance* Accommodation WAS

25. I was too trusting Assimilation Created

26. I should have trusted my gut feeling Assimilation Created

27. I must have been punished for something I did Assimilation Created

28. The event happened to me because of the sort of person I am Assimilation PTCI

29. Somebody else would have stopped the event from happening Assimilation PTCI

30. Somebody else would not have gotten into this situation Assimilation PTCI

31. There is something about me that made the event happen Assimilation PTCI

32. If the person really cared about me that person would not have done what they did Assimilation TAQ

33. Someone important (such as a parent, lover, friend) should have kept me safe Assimilation TAQ

34. I was not responsible for what happened to me*+ Assimilation TAQ

35. If I were good enough, then this wouldn’t have happened to me Assimilation TAQ

36. I deserved what happened to me Assimilation TAQ

37. I let myself down Assimilation TAQ

38. I must have done something really awful to make this happen Assimilation TAQ

39. I had some thoughts or beliefs that I should not have had Assimilation TRGI

40. I should have had certain feelings that I did not have Assimilation TRGI

41. I had good reasons for doing what I did+ Assimilation TRGI

42. What I did made sense+ Assimilation TRGI

43. If I knew today only what I knew when the event occurred,

I would do exactly the same thing+ Assimilation TRGI

44. What I did was completely justified+ Assimilation TRGI

45. I have reason to be ashamed of my personal character Assimilation WAS

46. Misfortune is least likely to strike worthy, decent people Assimilation WAS

47. Generally, people deserve what they get in this world Assimilation WAS

48. People’s misfortunes result from mistakes they have made Assimilation WAS

49. Through our actions we can prevent bad things from happening to us Assimilation WAS

50. If people took preventative actions, most misfortune could be avoided Assimilation WAS

51. When bad things happen, it is typically because people have not taken the

necessary actions to protect themselves Assimilation WAS

52. I am not confident that I can make good decisions for myself * Overaccommodation PBMS

53. It is possible for me to have close and loving feelings with other people+ Overaccommodation PMBS

54. I can’t stop bad things from happening Overaccommodation PTCI

55. I can’t rely on myself Overaccommodation PTCI

56. People can’t be trusted Overaccommodation PTCI

57. I have to be on guard all the time Overaccommodation PTCI

58. I have to be especially careful because you never know what will happen next Overaccommodation PTCI

59. The world is a dangerous place Overaccommodation PTCI

60. I can’t rely on other people Overaccommodation PTCI

61. People are not what they seem Overaccommodation PTCI

62. People are naturally unfriendly and unkind Overaccommodation WAS

63. People don’t really care what happens to the next person Overaccommodation WAS

64. I trust my own judgment+ Overaccommodation WAS

65. I lost a piece of myself Overaccommodation TAQ

____________________________________________________________________________________________________________

Note. PTCI: Posttraumatic Cognitions Inventory (Foa et al., 1999). TAQ: Trauma Appraisal Questionnaire (DePrince et al., 2010). PMBS: Posttraumatic Maladaptive Beliefs Scale (Resick et al., 2010). TRGI: Trauma-Related Guilt Inventory (Kubany et al., 1996). WAS: World Assumptions Scale (Janoff-Bulman, 1989). *Re-worded from original item: #37. I don’t feel confident that I can make good decisions for myself, #42. I was responsible for what happened to me, #61. The course of our lives is largely determined by chance, #62. Life is too full of uncertainties that are determined by chance, #74. Danger was always present, #75. No shower could wash away how dirty I felt, #77. It’s as if my insides were dirty, #78.
