# Supplementary material for: Development and validation of the Trauma-Related Cognitions Scale
Source: PLoS One. 2021 Apr 15;16(4):e0250221. doi: 10.1371/journal.pone.0250221 (PMC8049256; doi:10.1371/journal.pone.0250221)
Supplement: S2 Appendix — (DOCX) [file pone.0250221.s002.docx]

**S2 Appendix. Trauma-Related Cognitions Scales.**

We are interested in the kind of thoughts which you may have had after a traumatic experience. Below are a number of statements that may or may not be representative of your thinking.

Please read each statement carefully and tell us how much you AGREE or DISAGREE with each statement using the following rating scale. People react to traumatic events in many different ways. There are no right or wrong answers to these statements.

*Strongly Disagree Somewhat Somewhat Agree Strongly*

*Disagree Disagree Agree Agree*

1 2 3 4 5 6

01. I can trust my friends

02. I have no future

03. My life has been destroyed by the trauma

04. I knew better than to do what I did

05. The good things that happen in this world far outnumber the bad

06. I have made good and bad choices in life

07. Human nature is basically good

08. This event(s) could have been avoided

09. You never know when something terrible will happen

10. By and large, good people get what they deserve in this world

11. Some people can be trusted

12. I am a weak person

13. I am inadequate

14. I will get upset if someone pushes me too far

15. I am very satisfied with the kind of person I am

16. I blame myself for what happened

17. I did something that went against my values

18. Most people are basically caring

19. My reactions since the event mean that I am going crazy

20. It would not have happened if I would have been paying attention

21. I have lost my sense of freedom

22. I am a bad person

23. I should have known better

24. I will not be able to control my emotions, and something terrible will happen

25. Important people (such as parents, partner, friend) let this happen to me

26. It’s as if my insides are dirty

27. I can’t deal with even the slightest upset

28. People are basically kind and helpful

29. My emotions are typical of most people

30. Other people can be genuinely loving toward me

31. I hold myself responsible for what happened

32. Nothing good can happen to me anymore

33. Life is sometimes a gamble

34. If I think about the event, I will not be able to handle it

35. People will experience good fortune if they themselves are good

36. Sometimes bad things happen for no good reason

37. I can’t trust that I will do the right thing

38. What I did was inconsistent with my beliefs

39. You can never know who will harm you

40. If you look closely enough, you will see that the world is full of goodness

41. I used to be a happy person but now I am always miserable

42. I did the best I could in an unpredictable situation

43. I have permanently changed for the worse

44. The event happened because I wasn’t careful enough

45. Life is about surviving challenging events

46. Most people are capable of good things

47. There is something wrong with me as a person

48, I have made some mistakes, but that does not make me a bad person

49. I am not safe

50. The world has good and bad people in it

51. I will never be able to feel normal emotions again

52. The event happened because of the way I acted

53. There is more good than evil in this world

54. My reactions since the trauma show that I am a lousy coper

55. I could have prevented what happened to me

56. I lost my sense of manhood or womanhood

57. I blame myself for something I did, thought, or felt

58. Sometimes good people do bad things

59. I had some feelings that I should not have had

60. No shower can wash away how dirty I feel

61. I will not be able to control my anger and will do something terrible

62. Overall, I am a good person despite some of my faults

63. Danger is always present

64. I have lost respect for myself

65. I will not be able to tolerate my thoughts about the event, and I will fall apart

66. I comfort myself very well when I’m upset

67. One cannot always predict the outcome of a situation

68. The world is a good place

69. Sometimes bad things happen to good people

**Scoring Algorithm:** To compute the subscales, sum the items within each subscale and divide by the number of items to compute mean scale-score values.

Overaccommodation items: mean (2, 3, 12, 13, 19, 21, 22, 24, 25, 26, 27, 32, 34, 37, 41, 43, 47, 49, 51, 54, 56, 60, 61, 64, 65).

Assimilation items: mean (4, 8, 16, 17, 20, 23, 31, 38, 44, 52, 55, 57, 59).

Accommodation items: mean (6, 9, 14, 33, 36, 39, 42, 45, 48, 50, 58, 62, 63, 67, 69).

Optimism items: mean (1, 5, 7, 10, 11, 15, 18, 28, 29, 30, 35, 40, 46, 53, 66, 68).
